# *C. elegans mlt-11* activity is necessary in seam cells for molting and larval development

**DOI:** 10.17912/micropub.biology.002135

**Published:** 2026-05-19

**Authors:** James Matthew Ragle, Jordan D. Ward

**Affiliations:** 1 Department of Molecular, Cell, and Developmental Biology, University of California – Santa Cruz, Santa Cruz, CA 95064

## Abstract

*C. elegans* gene *mlt-11* is a Kunitz family putative protease inhibitor required for proper cuticle formation and molting. Using tissue-specific RNA interference we tested in which tissues *mlt-11* activity was critical for molting and developmental progression. *mlt-11* knockdown using a seam cell-enriched RNAi strain produced similar phenotypes as knockdown throughout animals. In contrast, hypodermis-enriched knockdown caused no detectable phenotype. Together, our findings identify seam cells as a critical site of *mlt-11* function and highlight an unexpectedly central role for this specialized epithelial cell type in regulating molting and developmental progression.

**Figure 1. ​​ mlt-11 is necessary in seam cells for molting and larval development:** (A) Tissue-specific RNAi strains used. A timed egg lay of animals of the indicated genotype was performed on control or *mlt-11 (RNAi)* plates and phenotypes were scored three days later. (B) Representative images of animals of the indicated genotype grown on control or *mlt-11* RNAi. The red box highlights conditions that produced smaller larvae with molting defects. (C) Developmental delay was scored in the indicated strains grown on control or *mlt-11* RNAi plates and classified as a failure to reach adulthood after 72 hours of growth. (D) Molting defects were scored in the indicated strains on control or *mlt-11* RNAi. Scored defects included animals dragging cuticles, ecdysis failure and cuticle corsets. Tissue-specific RNAi data is from two independent replicates and the number of animals scored is in (A).

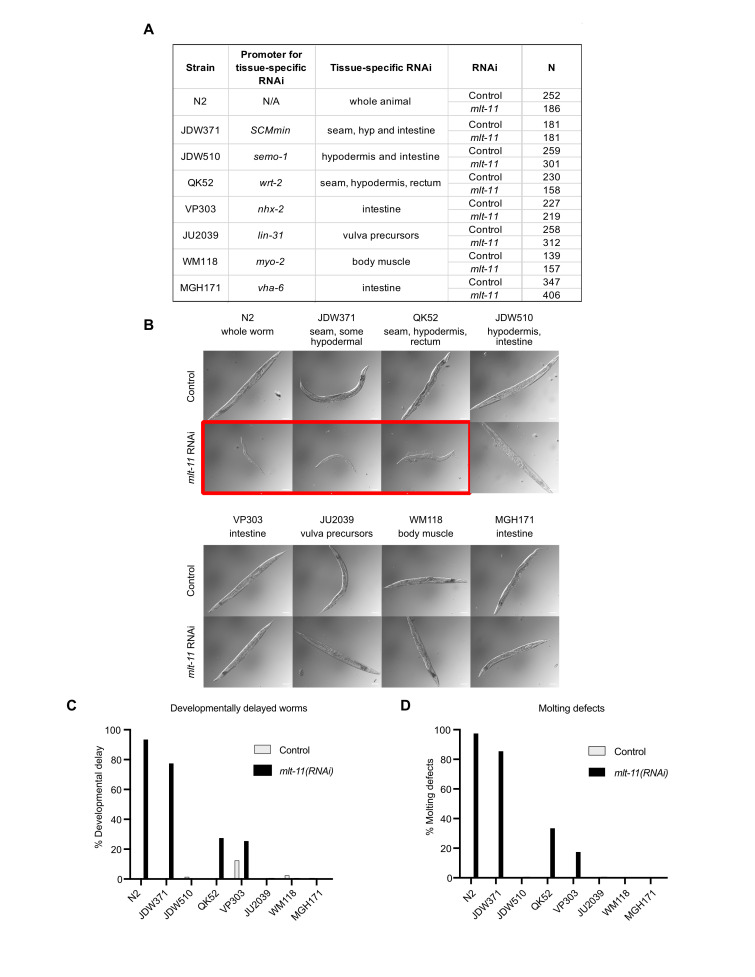



## Description

Molting is an essential developmental process in nematodes, involving periodic synthesis of a new collagen-rich apical extracellular matrix (cuticle) and the shedding of the old cuticle. Understanding the mechanisms underlying molting can provide insight into oscillatory biological timers, epithelial biology, and is a source of potential targets to develop anthelminthics to control parasitic nematodes of medical and agricultural importance. Proteases and protease inhibitors play important and poorly understood roles in remodeling of the *C. elegans* aECM during molting.

*mlt-11* is a putative Kunitz family protease inhibitor that plays a critical role in *C.elegans* cuticle synthesis and molting (Frand et al., 2005; Ragle et al., 2026). It is a complex protein with 10 Kunitz domains and depending on which of these domains are deleted, phenotypes such as left rollers, right rollers, microblistering, or embryonic lethality are observed (Ragle et al., 2026). Reduction of *mlt-11* function results in aberrant protein localization in the aECM over both seam and hypodermal cells, and all three layers of the adult cuticle were affected (basal, medial, and cortical layers) (Ragle et al., 2026). Given that *mlt-11* is expressed in both seam cells and in the hypodermal syncytium (Frand et al., 2005; Ragle et al., 2026), we turned to a tissue-specific RNAi approach&nbsp; to test where *mlt-11* activity was needed to promote molting ( Fig. 1A ). *mlt-11* knockdown in a tissue-specific RNAi strain ( JDW371 ) that restricts knockdown to seam, hypodermal, and intestinal cells (Johnson et al., 2023), phenocopied *mlt-11* RNAi in wild-type animals with respect to developmental delay and molting defects ( Fig. 1B- D). *mlt-11* RNAi in a hypodermal and seam cell-specific RNAi strain ( QK52 ), produced less penetrant developmental delay and molting defects ( Fig. 1B- D). Notably, *mlt-11* RNAi in a hypodermal and intestinal-specific RNAi strain ( JDW510 ), produced no developmental delay or molting defects, suggesting *mlt-11* activity is necessary in seam cells and perhaps, in a limited capacity, in other hypodermal cells ( Fig. 1B- D).

Despite robust hypodermal expression and localization (Frand et al., 2005; Ragle et al., 2026), our tissue-specific RNAi suggests that the seam cells are an important site of *mlt-11* expression (Fig. 1). We consistently saw the most severe RNAi phenotypes in tissue-specific RNAi strains using a seam cell enriched promoter and no phenotypes from a hypodermal/intestinal-specific RNAi strain ( Fig. 1B- D). Consistent with these data, we previously observed aberrant expression of the markers DPY-7 ::GFP (furrows), DPY-10 ::mScarlet (furrows), ROL-6 ::mNG (basal layer collagen), CUT-2 ::mNG (cortical layer cuticlin), and COL-19 ::mNG (basal layer collagen) over the seam cells as well as alae gaps following *mlt-11* inactivation (Ragle et al., 2026). The medial layer strut collagen BLI-1 is normally excluded from the aECM over the seam cells but invades this area following *mlt-11* knockdown (Ragle et al., 2026). These data were reminiscent of our work on NHR-23 as depletion of this factor causes aberrant aECM formation over the seam cells and *nhr-23* activity also was necessary in the seam cells for developmental progression (Johnson et al., 2023). Seam cells are responsible for the production of alae (Singh & Sulston, 1978), so it is unclear why inactivation of *nhr-23* or *mlt-11* in the seam cells has such a potent effect on developmental progression and molting given that the hypodermis secretes the bulk of the cuticle. *mlt-11* inactivation has been shown to cause reduced body length and width, impaired cuticle barrier function, and aberrant aECM protein localization (Ragle et al., 2026). Defining the tissue-specific requirements for *mlt-11* in preventing these defects will be an important avenue for future investigation.

## Methods

**RNAi Knockdown**

RNA interference experiments were performed as in Johnson et al. (2023). Control RNAi used an empty L4440. The *mlt-11 (RNAi)* vector was streaked from the Ahringer library (Kamath et al., 2003).&nbsp;

## Reagents

**Table:** 

**Strain name**	**Genotype**	**Source**
N2	Bristol isolate, standard lab wild type	CGC
JDW371	*jsTi1493 {mosL loxP [ wrdSi72 (SCMmin:: rde-1 CDS+3'UTR)] FRT3::mosR} IV ; rde-1 ( ne300 ) V *	Ward lab (Johnson et al., 2023)
JDW510	*jsTi1493 {mosL loxP [ wrdSi97 (semo-1p:: rde-1 CDS+3'UTR)] FRT3::mosR} IV ; rde-1 ( ne300 ) V *	Ward lab (Johnson et al., 2023)
JU2039	*mfIs70 [lin-31p:: rde-1 + myo2p::GFP] IV ; rde-1 ( ne219 ) V *	CGC (Barkoulas et al., 2013)
MGH171	*alxIs9 [vha-6p:: sid-1 ::SL2::GFP] sid-1 ( qt9 ) V ; alxIs9*	CGC (Melo & Ruvkun, 2012)
QK52	*rde-1 ( ne219 ) V ; xkIs99 (wrt-2p:: rde-1 :: unc-54 3'UTR) *	CGC (Melo & Ruvkun, 2012)
WM118	*rde-1 ( ne300 ) V ; neIs9 ( neIs9 [ myo-3 ::HA:: RDE-1 + rol-6 ( su1006 )]) X *	CGC (Watts et al., 2020)
VP303	*rde-1 ( ne219 ) V ; kbIs7 [nhx-2p:: rde-1 + rol-6 ( su1006 )]) *	CGC (Espelt et al., 2005)
